# Targeting Hsp90 with small molecule inhibitors induces the over-expression of the anti-apoptotic molecule, survivin, in human A549, HONE-1 and HT-29 cancer cells

**DOI:** 10.1186/1476-4598-9-77

**Published:** 2010-04-15

**Authors:** Chun Hei Antonio Cheung, Huang-Hui Chen, Li-Ting Cheng, Kevin W Lyu, Jagat R Kanwar, Jang-Yang Chang

**Affiliations:** 1 National Institute of Cancer Research, National Health Research Institutes (NHRI), Tainan 70456, Taiwan ROC; 2 Department of Biological Science, University of Southern California, California, USA; 3 Institute of Biotechnology (BioDeakin), Institute for Technology Research and Innovation, Deakin University, Geelong, Victoria 3217, Australia; 4 Division of Hematology and Oncology, Department of Internal Medicine, National Cheng Kung University Hospital, Tainan 70456, Taiwan ROC

## Abstract

**Background:**

Survivin is a dual functioning protein. It inhibits the apoptosis of cancer cells by inhibiting caspases, and also promotes cancer cell growth by stabilizing microtubules during mitosis. Since the molecular chaperone Hsp90 binds and stabilizes survivin, it is widely believed that down-regulation of survivin is one of the important therapeutic functions of Hsp90 inhibitors such as the phase III clinically trialed compound 17-AAG. However, Hsp90 interferes with a number of molecules that up-regulate the intracellular level of survivin, raising the question that clinical use of Hsp90 inhibitors may indirectly induce survivin expression and subsequently enhance cancer anti-drug responses. The purpose of this study is to determine whether targeting Hsp90 can alter survivin expression differently in different cancer cell lines and to explore possible mechanisms that cause the alteration in survivin expression.

**Results:**

Here, we demonstrated that Hsp90 inhibitors, geldanamycin and 17-AAG, induced the over-expression of survivin in three different human cancer cell lines as shown by Western blotting. Increased survivin mRNA transcripts were observed in 17-AAG and geldanamycin-treated HT-29 and HONE-1 cancer cells. Interestingly, real-time PCR and translation inhibition studies revealed that survivin was over-expressed partially through the up-regulation of protein translation instead of gene transcription in A549 cancer cells. In addition, 17-AAG-treated A549, HONE-1 and HT-29 cells showed reduced proteasomal activity while inhibition of 26S proteasome activity further increased the amount of survivin protein in cells. At the functional level, down-regulation of survivin by siRNA further increased the drug sensitivity to 17-AAG in the tested cancer cell lines.

**Conclusions:**

We showed for the first time that down-regulation of survivin is not a definite therapeutic function of Hsp90 inhibitors. Instead, targeting Hsp90 with small molecule inhibitors will induce the over-expression of survivin in certain cancer cell lines and subsequently enhances the ability of cell survival in drug-treated situations. The current study suggests that dual inhibition of Hsp90 and survivin may be warranted.

## Introduction

Heat shock protein 90 (Hsp90) is a molecular chaperone that assists the correct folding and stabilization of various proteins in cells. During the last decade, Hsp90 has emerged as an exciting target for cancer therapy. The over-expression of Hsp90 has been shown in various cancers such as non-small cell lung cancer, oesophageal squamous cell carcinoma, pancreatic carcinoma and advanced malignant melanoma [1-4]. In addition, studies showed that Hsp90 stabilizes various key oncogenic proteins such as survivin, Akt, Erb-2 and HIF-1α in cancer cells [5-7]. Therefore, targeting hsp90 gives therapeutic advantages over other target-therapies as multiple Hsp90-related oncogenic proteins can be targeted simultaneously [7].

Survivin is a member of the inhibitors of apoptosis (IAPs) family. Unlike other IAPs, survivin is a bifunctional protein that functions as a key regulator of mitosis and inhibitor of programmed cell death. It is well-demonstrated that the over-expression of survivin induces resistance to various anti-cancer therapies such as chemotherapy and radiation therapy in cancer cells [8-12]. For example, over-expression of survivin has been shown to induce drug resistance against anti-mitotic compounds by stabilizing microtubule network in vincristine/colchicine-resistant oral cancer cells and down-regulation of it restores drug sensitivity to those compounds in the same cell line [9]. In addition, literature revealed that over-expression of survivin attenuated both tamoxifen and cisplatin-induced apoptosis in human breast cancer cells and gastric cancer cells respectively [10,12]. Interestingly, a recent report suggests that over-expression of survivin may also enhance DNA double-strand breaks (DBD) repair capability in radiation-treated oral cancer cells by up-regulating the molecular sensor of DNA damage, Ku70 [11]. In clinical situations, the level of survivin expression was shown to be inversely related to the levels of apoptosis and positively related to the risk of local tumor recurrence in rectal cancer patients treated with radiotherapy [13]. Furthermore, patients with gastric tumors that express lower level of survivin seems to have a longer mean survival time than patients with higher survivin expression level after cisplatin treatment [12]. It has also been shown that survivin expression is associated with human prostate cancer bone metastasis [14]. Thus, survivin plays an important role in tumorigenesis, tumor metastasis and may act as an indicator of therapeutic effectiveness.

It is widely believed that Hsp90 physically interacts and stabilizes survivin in cells [5,15,16]. Although Hsp90 is a molecular chaperone that assists the correct folding of various proteins in cells, it does not bind to unfolded survivin [5]. Instead, Hsp90 binds to the mature form of survivin [5]. Structurally, the amino acid sequence Lys-70-Lys-90 of survivin is important for the binding to the N-terminal domain (ATP-binding site) of Hsp90 [5]. Various studies have investigated the possibility of targeting survivin using Hsp90 inhibitors, based on the fact that survivin is important for cancer survival and progression. Hsp90 inhibitors such as geldanamycin, 17-AAG and shepherdin have been shown effective in targeting the Hsp90/survivin complex and subsequently inducing proteasomal degradation of survivin [5,16-18].

Although it is widely believed that Hsp90 inhibitors induce cancer cell death through indirect down-regulation of survivin as one of its multiple therapeutic functions, a study demonstrated that 17-AAG treatment slightly increased the amount of survivin present in the human DU145 prostate cancer cells [7]. However, the mechanism of the over-expression of survivin in such cell line was unknown. Interestingly, we also observed an up-regulation of survivin in 17-AAG and geldanamycin-treated human A549, HONE-1 and HT-29 cancer cells. Since Hsp90 interferes with multiple molecules such as sp1, sp3 (both transcriptional factors of survivin), and 26S proteasome (negative regulator of survivin protein level) simultaneously [19,20], we hypothesize that targeting Hsp90 will affect the expression of survivin at various stages. We also hypothesize that the use of Hsp90 inhibitors may not be able to down-regulate survivin expression in certain cancer cells. Therefore, the purpose of this study is to determine whether targeting Hsp90 can alter survivin expression differently in different cancer cell lines and to explore possible mechanisms that cause the alteration in survivin expression.

## Results

### Targeting Hsp90 induces the over-expression of survivin in cancer cells

**Figure 1:**
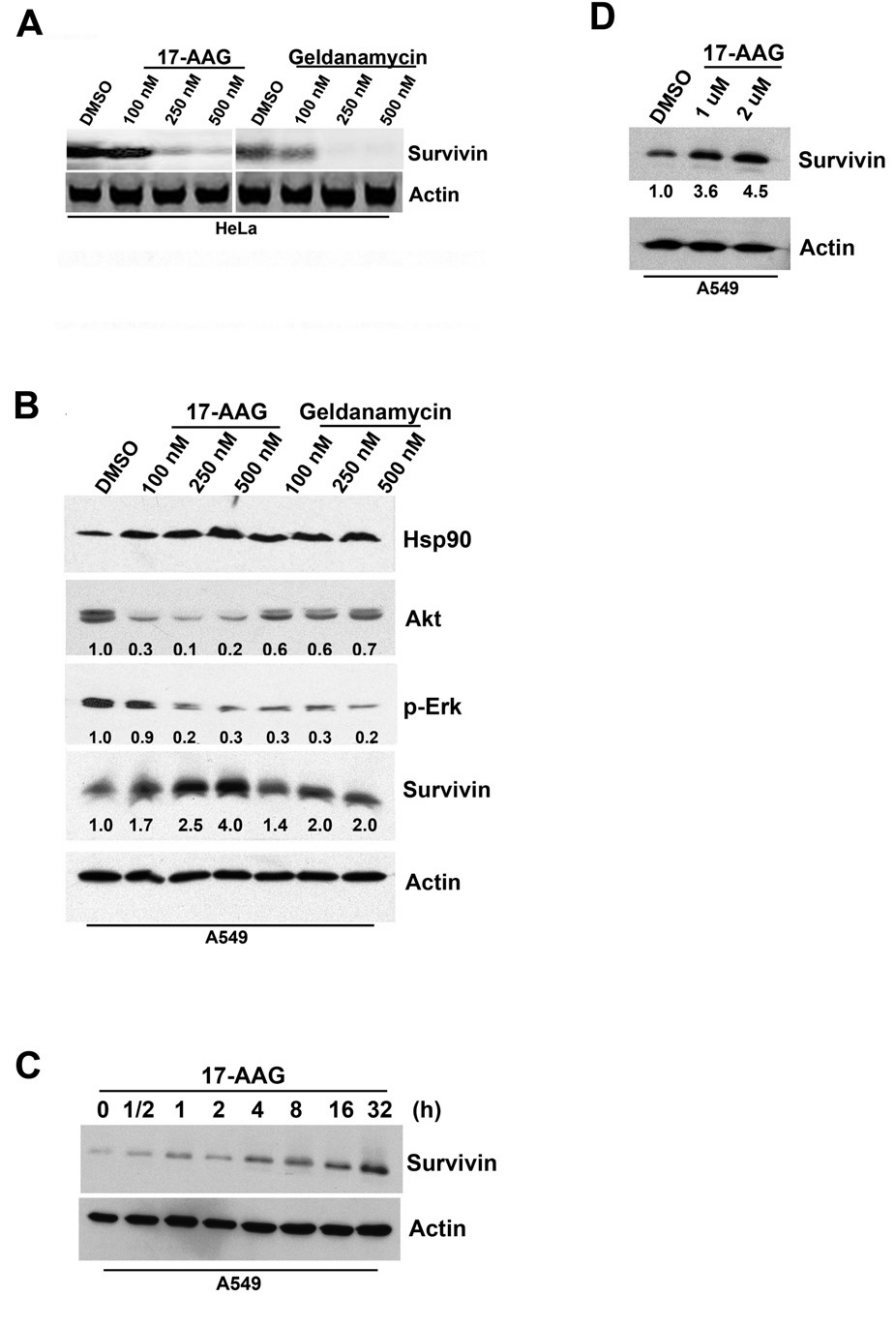
**Inhibition of Hsp90 with small molecule inhibitors induces survivin over-expression in human A549 cancer cells**. (**A**) Concentration-dependent down-regulation of survivin in drug-treated human HeLa cancer cells. HeLa cells were treated with DMSO or various concentrations (100 nM, 250 nM and 500 nM) of 17-AAG and geldanamycin for 24 hours. Cell lysate was prepared after the treatment and various proteins were resolved by SDS-PAGE. (**B**) Concentration-dependent over-expression of survivin in drug-treated human A549 cancer cells. A549 cells were treated with DMSO or various concentrations (100 nM, 250 nM and 500 nM) of 17-AAG and geldanamycin for 24 hours. Cell lysate was prepared after the treatment and various proteins were resolved by SDS-PAGE. The expression of Hsp90, Akt, phosphorylated-Erk and survivin was revealed by Western blot analysis. Actin was used as an internal control. Relative ratios of expression were shown. (**C**) Time-dependent over-expression of survivin in drug-treated human A549 cancer cells. Human A549 cells were treated with 500 nM of 17-AAG for various intervals. Cell lysate was prepared after the treatment and various proteins were resolved by SDS-PAGE. The expression of survivin was revealed by Western blot analysis. Actin was used as an internal control. (**D**) Over-expression of survivin in human A549 cells treated with high concentrations of 17-AAG. A549 cells were treated with 1-2 μM of 17-AAG for 24 hours. Cell lysate was extracted after the treatment and various proteins were resolved by SDS-PAGE. Relative ratios of expression were shown.

To determine whether the inhibition of Hsp90 with small molecule inhibitors was able to affect survivin expression, we treated human cancer cells with Hsp90 inhibitors geldanamycin and 17-AAG. 17-AAG is a selective Hsp90 inhibitor that exhibited therapeutic activities in various cancers and is currently undergoing phase III clinical trials [17,21-24]. To ensure that both of our selected Hsp90 inhibitors were functioning normally at the molecular level, HeLa cells (positive control) were incubated with 17-AAG and geldanamycin for 24 h and Western blot analysis was used to determine the amount the survivin presented in cells. Consistent with other studies, targeting Hsp90 with 17-AAG and geldanamycin (100 nM, 250 nM and 500 nM) reduced the amount of survivin expressed in HeLa cells (Figure 1A) [5]. The effectiveness of Hsp90 inhibitors was subsequently tested with the use of the three-dimensional cell culturing system. Western blot analysis revealed that the expression of survivin was also down-regulated in three-dimensionally cultured HeLa cells treated with 1 μM of 17-AAG for 24 h (Additional file 1).

To determine whether Hsp90 inhibitors were able to down-regulation survivin in other cancer cell lines, human A549, HT-29 and HONE-1 cancer cells were used. In A549 cells, targeting Hsp90 with 17-AAG and geldanamycin slightly induced the baseline expression of Hsp90 as previously reported (Figure 1B) [5]. The same treatment also induced down-regulation of both Akt and phosporylated-Erk in human A549 (p53-mutant) lung carcinoma cells as expected (Figure 1B) [7,25]. Together, these results indicated that both Hsp90 inhibitors were functioning normally at the molecular level. Surprisingly, targeting Hsp90 with 17-AAG and geldanamycin did not induce survivin down-regulation in A549 cancer cells. Instead, Western blot analysis revealed that survivin expression was induced by Hsp90 inhibitors in A549 cells in a concentration-dependent manner (Figure 1B). In addition, over-expression of survivin was shown in cells treated with 17-AAG in a time-dependent manner (Figure 1C). Since clinical study of 17-AAG revealed that the maximum peak serum level of this compound could reach 2-3 μM, A549 cells were further treated with high concentrations of 17-AAG and the expression of survivin was determined. Over-expression of survivin was also found in A549 cells treated with high concentrations (1 μM and 2 μM) of 17-AAG (Figure 1D). Moreover, Western blot analysis revealed that the expression of survivin was up-regulated in three-dimensionally cultured A549 cells treated with 1 μM of 17-AAG for 24 h (Additional file 1).

**Figure 2:**
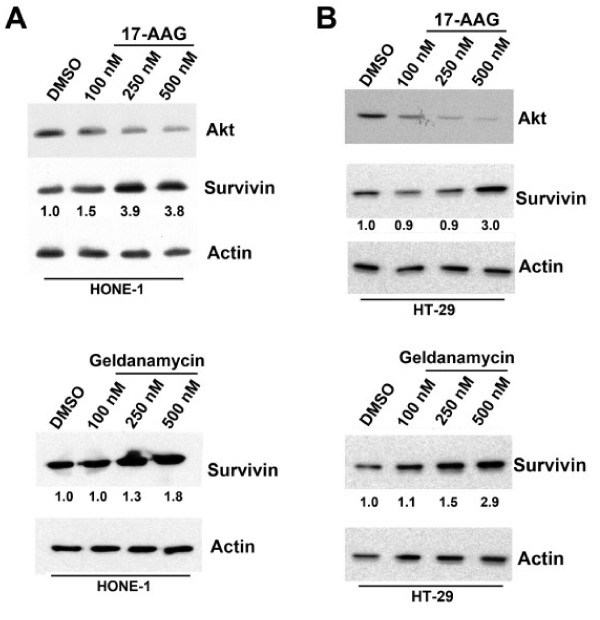
**Over-expression of survivin in various 17-AAG/geldanamycin-treated human cancer cell lines**. **(A) **Human HONE-1 cancer cells were treated with various concentrations of 17-AAG/geldanamycin for 24 hours. Cell lysate was prepared after the treatment and various proteins were resolved by SDS-PAGE. The expression of Akt and survivin was revealed by Western blot analysis. Actin was used as an internal control. Relative ratio of expression was shown. (**B**) Human HT-29 cancer cells were treated with various concentrations of 17-AAG/geldanamycin for 24 hours. Cell lysate was prepared after the treatment and various proteins were resolved by SDS-PAGE. The expression of Akt and survivin was revealed by Western blot analysis. Actin was used as an internal control. Relative ratio of expression was shown.

The expression of survivin in Hsp90 inhibitors-treated HONE-1 and HT-29 cells was determined by Western blotting. Western blot analysis revealed that both 17-AAG and geldanamycin treatments were able to induce the over-expression survivin in HONE-1 (p53 wildtype) nasopharyngeal carcinoma cells in a concentration-dependent manner (Figure 2A). 17-AAG or geldanamycin treated HT-29 (p53 mutant) colon adenocarcinoma cells also over-expressed survivin (Figure 2B). In contrast, 17-AAG treatment was able to reduce the expression of Akt in a concentration-dependent manner as previously reported (Figures 2A and 2B) [7,25]. Taken together, these data indicate that the surprising effect of Hsp90 inhibitors were specific to survivin in this study, as 17-AAG and geldanamycin did not modulate the expression of Hsp90, Akt and phosphorylated-Erk in A549 cells and the expression of Akt in HONE-1 and HT-29 cells differently compared to the results of other studies. In three-dimensional culture situations, Western blot analysis revealed that 17-AAG treatment increased the amount of survivin presented in HONE-1 and HT-29 cells (Additional file 1). These results suggest that down-regulation of survivin is not a definite therapeutic effect induced by Hsp90 inhibitors, as over-expression of survivin was observed in cells treated with 17-AAG and geldanamycin in this study.

### Targeting Hsp90 induces the over-expression of survivin through a cell-cycle independent mechanism

**Figure 3:**
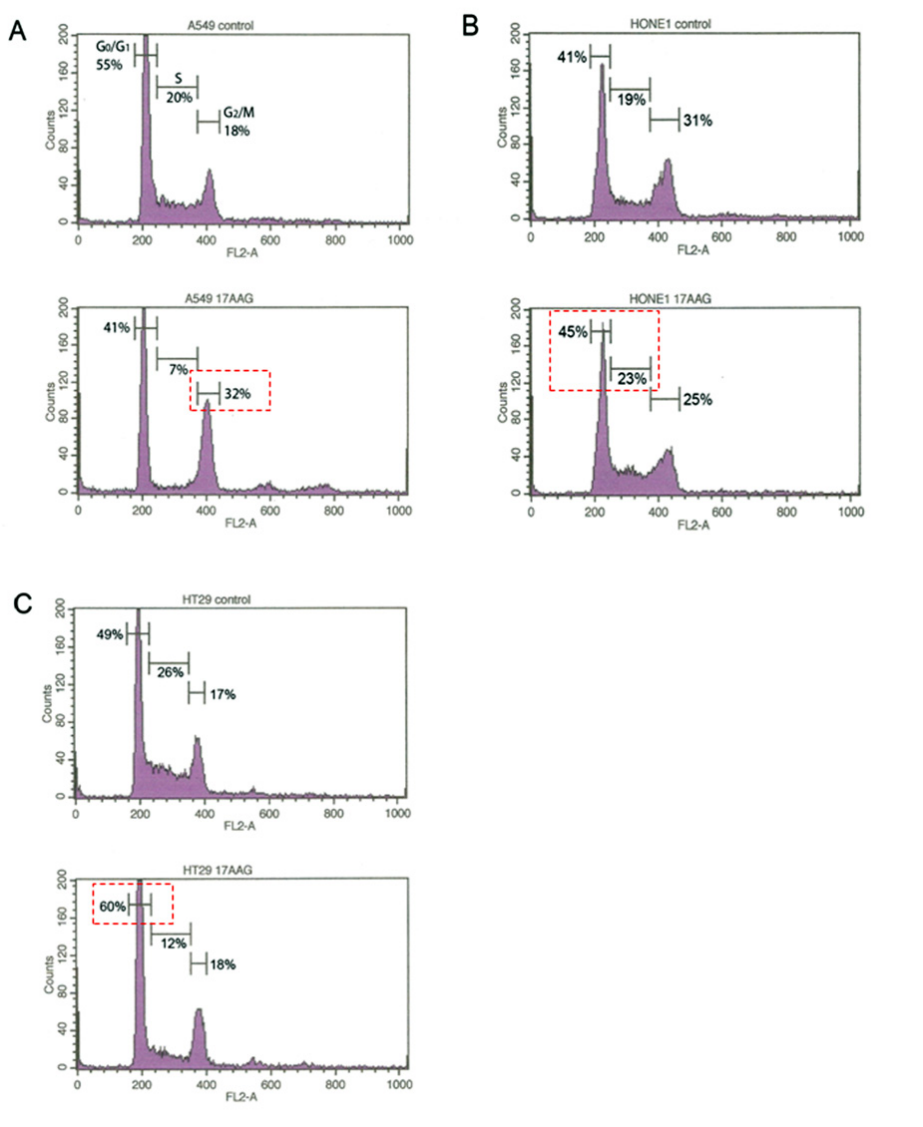
**17-AAG induces the over-expression of survivin through cell cycle-independent mechanism**. A549 **(A)**, HONE-1 **(B) **and HT-29 **(C) **cells treated with 500 nM of 17-AAG for 24 h were stained with propidium iodide and subsequent analyzed by flow cytometry. Major changes in the cell cycle of 17-AAG-treated cells were highlighted in red color.

It has been widely demonstrated that the expression of survivin is tightly regulated during cell cycle and maximized during the G_2_/M phase. To determine whether the over-expression of survivin in Hsp90-targeted cells was a downstream result caused by cell cycle arrest at the G_2_/M phase, flow cytometric analysis was performed. Interestingly, 17-AAG treatment did not induce a uniform cell cycle response among A549, HONE-1 and HT-29 cells. Experimental results demonstrated that 500 nM of 17-AAG induced cell cycle arrest of A549 cells at the G_2_/M phase after 24 h (Figure 3). In contrast, the same treatment induced S-phase arrest and G_0_/G_1 _phase cell cycle arrest in HONE-1 and HT-29 cells respectively (Figure 3). Taken together, results from both Western blot analysis and flow cytometric analysis indicated that changes in survivin expression in Hsp90-targeted cells were not downstream results caused by cell cycle arrest at the G_2_/M phase.

### Targeting Hsp90 affects survivin expression at the transcriptional level

**Figure 4:**
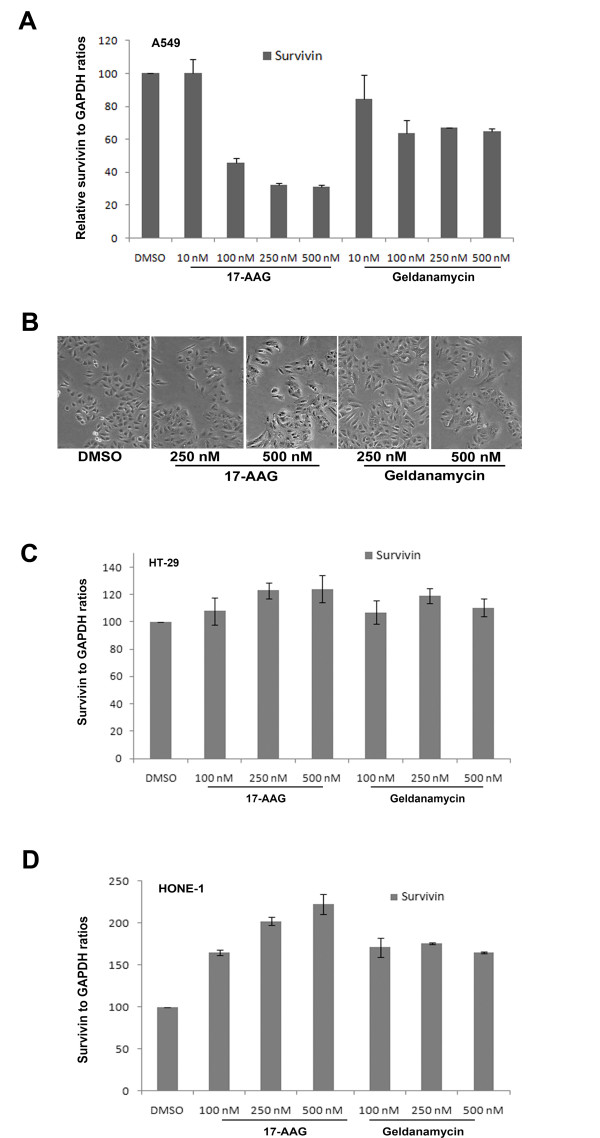
**17-AAG treatment induces over-expression of survivin through transcription-independent mechanisms**. **(A) **Inhibition of Hsp90 reduced the amount of survivin mRNA transcript presented in 17-AAG treated A549 cells. Human A549 cancer cells were incubated with DMSO (negative control) or various concentrations (10 nM, 100 nM, 250 nM and 500 nM) of 17-AAG and geldanamycin for 24 hours. Quantitative RT-PCR was performed to determine the amount of survivin transcripts presented in cells. (**B**) Cells treated with 17-AAG and geldanamycin did not induce abnormal cell morphology. A549 cells incubated with various concentrations (250 nM and 500 nM) of 17-AAG and geldanamycin for 24 hours and cell morphology was revealed by phase-contrast microscopy. (**C**) Inhibition of Hsp90 increased the amount of survivin mRNA transcript presented in 17-AAG and geldanamycin treated HT-29 cells. HT-29 cells were incubated with DMSO (negative control) or various concentrations (10 nM, 100 nM, 250 nM and 500 nM) of 17-AAG for 24 hours. Quantitative RT-PCR was performed to determine the amount of survivin transcripts presented in cells. (**D**) Inhibition of Hsp90 increased the amount of survivin mRNA transcript presented in 17-AAG treated HONE-1 cells. HONE-1 cells were incubated with DMSO (negative control) or various concentrations (10 nM, 100 nM, 250 nM and 500 nM) of 17-AAG for 24 hours. Quantitative RT-PCR was performed to determine the amount of survivin transcripts presented in cells.

Since 17-AAG induced over-expression of survivin was not an indirect result caused by cell cycle arrest, detailed molecule mechanisms that govern survivin over-expression were investigated. It is widely believed that the amount of protein presented in cells is tightly regulated through the process of gene transcription, protein translation and protein degradation. To determine whether the over-expression of survivin in Hsp90 inhibitor-treated A549, HONE-1 and HT-29 cells was caused by changes at the level of gene transcription, quantitative real-time PCR was performed after 24 h post-treatment. In contrast to the result of Western blot analysis, a dose-dependent decrease in the amount of survivin mRNA transcript in 17-AAG-treated A549 cells was shown by real-time PCR (Figure 4A). A general decrease in the amount of survivin mRNA transcript was also shown in geldanamycin-treated cells (Figure 4A). Cells treated with geldanamycin and 17-AAG showed reduced amount of survivin mRNA by 45% (500 nM) and 75% (500 nM) respectively in A549 cells, as compared to the control (Figure 4A). However, phase-contrast microscopy did not reveal morphological signs of death in Hsp90 inhibitors-treated cells (Figure 4B). Therefore, both geldanamycin and 17-AAG induced decreases in survivin mRNA instead of cell death-induced global decreases at the total intracellular mRNA level. These results also indicated that 17-AAG/geldanamycin treatment increased the level of survivin protein in cells possibly through transcription-independent mechanism in A549 cancer cells. In contrast, 17-AAG and geldanamycin treatment did not decrease the amount of survivin RNA transcript present in HT-29 and HONE-1 cells (Figure 4C and 4D). Instead, the same treatment increased the level of survivin RNA in cells in a dose-dependent manner. Thus, the effect induced by Hsp90 inhibitors on the level of survivin gene transcription seems to depend on the cellular context. Furthermore, the results suggest that increases in survivin protein in response to Hsp90-targeted therapy in HT-29 and HONE-1 cells are possibly due to increases in survivin gene transcription. Taken together, these results indicated that targeting Hsp90 with small molecule inhibitors might interfere with survivin gene transcription differently in different cancer cells.

### Targeting Hsp90 induces survivin expression through post-transcriptional mechanisms

**Figure 5:**
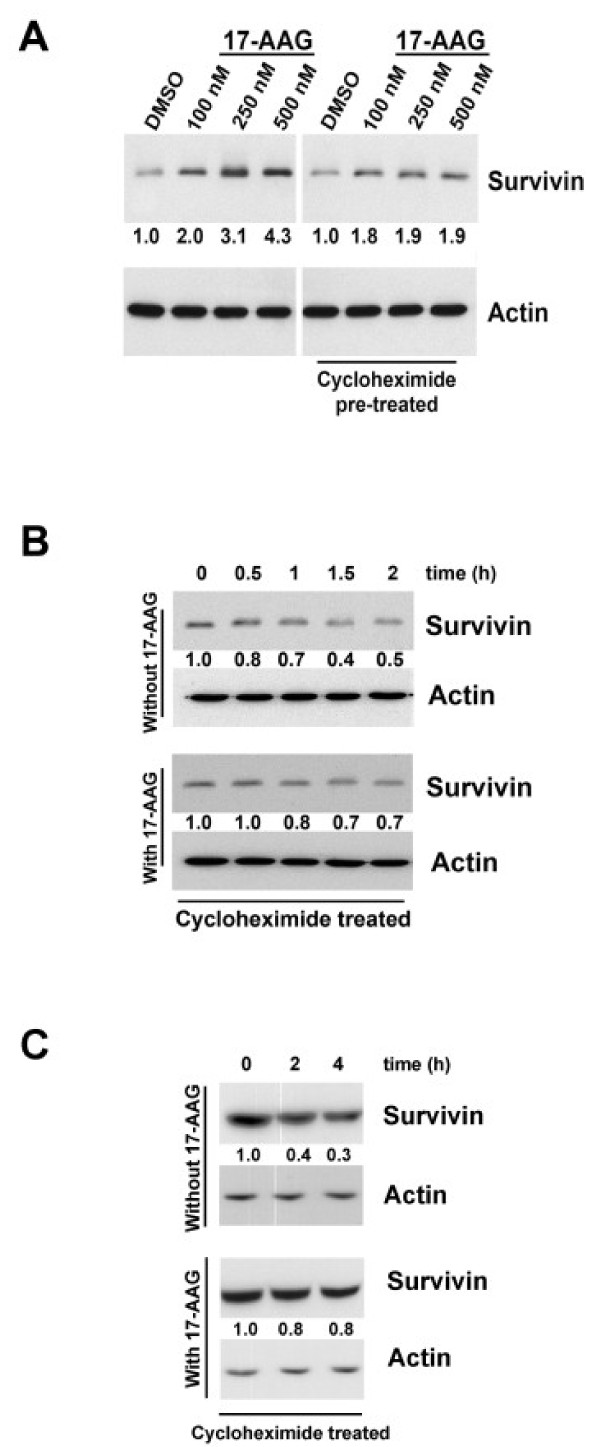
**Hsp90 inhibition induced over-expression of survivin through post-transcriptional mechanisms in A549 cells**. **(A) **Hsp90 inhibition induced over-expression of survivin partially through the process of protein translation. A549 cells were pre-incubated with/without 500 nM of cycloheximide for 1 hour and subsequently treated with DMSO or various concentrations (100 nM, 250 nM and 500 nM) of 17-AAG 24 hours. The expression of survivin was revealed by Western blot analysis. Actin was used as an internal control. Relative ratio of expression was shown. (**B**) 17-AAG reduced the rate of survivin protein degradation in A549 cells. A549 cells were pre-incubated with 500 nM of cycloheximide for 1 hour and subsequently treated with/without 250 nM of 17-AAG for various time. The expression of survivin was revealed by Western blot analysis. Actin was used as an internal control. Relative ratio of expression was shown. (**C**) The amount of survivin presented in un-treated A549 cells was less than that in 17-AAG-treated cells after 4 hours of protein synthesis inhibition. A549 cells were pre-incubated with 500 nM of cycloheximide for 1 hour and subsequently treated with/without 250 nM of 17-AAG for 2 hours and 4 hours. The expression of survivin was revealed by Western blot analysis. Actin was used as an internal control. Relative ratio of expression was shown.

Since the above results revealed that changes at the level of gene transcription did not contribute to the increase of survivin protein in 17-AAG treated A549 cells, possible post-transcriptional mechanisms such as protein translation and 26S proteasome-dependent protein degradation were investigated in this cell line. To determine whether 17-AAG induced over-expression of survivin through protein translation, translation inhibition study was performed. Human A549 cancer cells were pre-incubated with 500 nM of cycloheximide for three hours and subsequently treated with various concentrations of 17-AAG for 24 hours. Cycloheximide is a translational inhibitor that exerts its effect by interfering with the translocation step in protein synthesis, thus blocking translational elongation. Western blot analysis revealed dose-dependent increases of survivin in the 17-AAG-treated A549 cancer cells (Figure 5A). Interestingly, cycloheximide treatment completely abolished the dose-dependent pattern of survivin expression in 17-AAG-treated cells (Figure 5A). However, the amount of survivin in cycloheximide/17-AAG co-treated cells did not decrease to the baseline level of the negative control (Figure 5A). These results suggest that 17-AAG induces over-expression of survivin in A549 cancer cells partially through the regulation of protein translation. Moreover, other post-translational mechanisms may also contribute to the over-expression of survivin in 17-AAG-treated cells.

To determine whether 17-AAG may interfere with the stability of survivin protein in A549 cells, the rate of survivin protein degradation was determined in cells treated with/without 17-AAG. Cells were pre-incubated with 500 nM of cyclohexmide for one hour and subsequently co-incubated with 17-AAG for various time. Western blot analysis revealed that the degradation rate of survivin protein was slightly slower in 17-AAG treated A549 cells than the untreated cells (Figure 5B). The amount of survivin protein presented in 17-AAG untreated-cells was reduced by 70% after 4 h of protein synthesis inhibition (Figure 5C). In contrast, the amount of survivin presented in 17-AAG-treated cells was reduced by only 20% at the same time point (Figure 5C). Thus, the reduced rate of protein degradation might also contribute to the increased amount of survivin protein presented in 17-AAG treated A549 cancer cells.

### Targeting Hsp90 with 17-AAG reduces proteasomal activity in cancer cells

**Figure 6:**
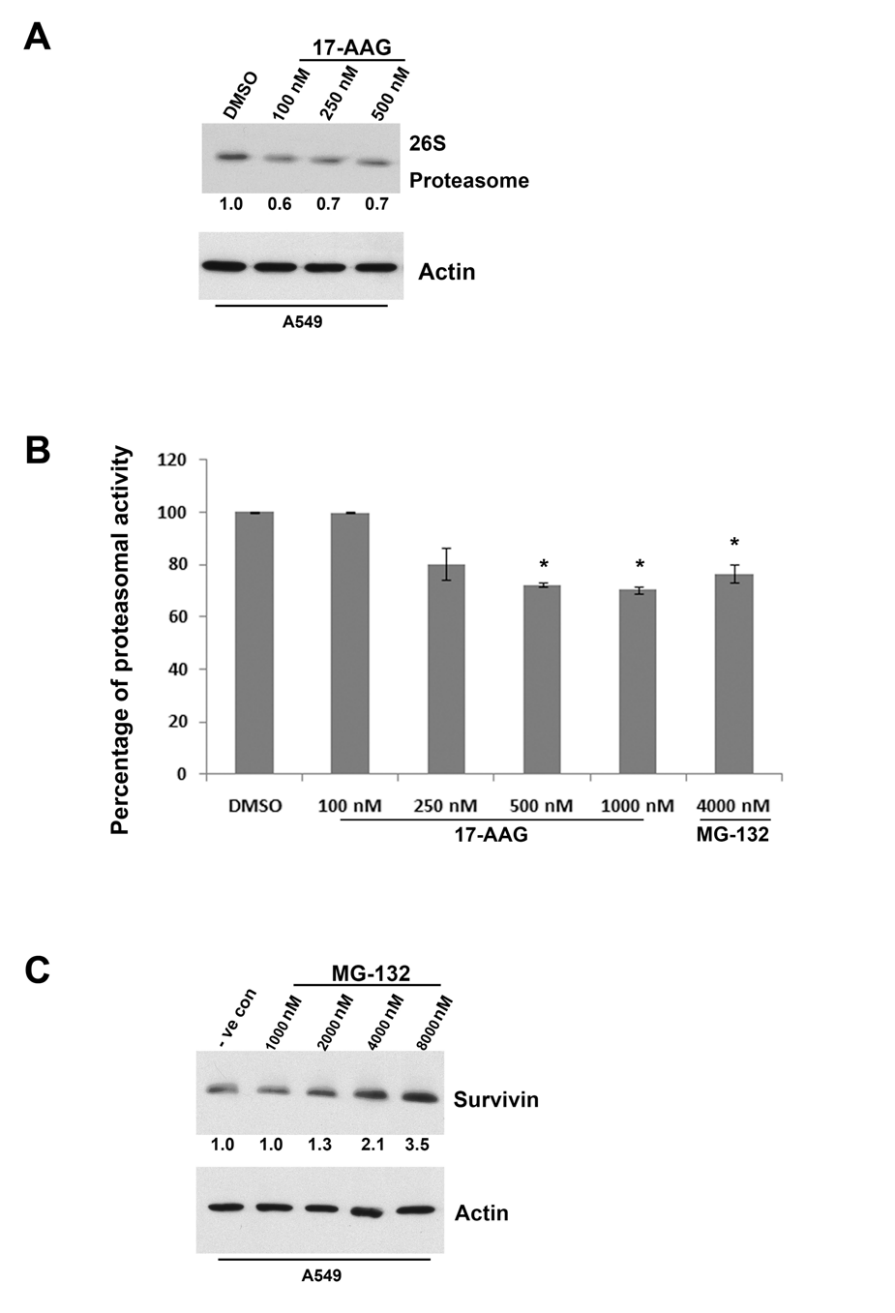
**17-AAG interfered with the amount and activity of 26S proteasome in A549 cells**. (**A**) 17-AAG treatment reduced the amount of 26S proteasome presented in A549 cells. A549 cells were treated with DMSO and various concentrations (100 nM, 250 nM and 500 nM) of 17-AAG for 24 hours. The expression of 26S proteasome was revealed by Western blot analysis. Actin was used as an internal control. Relative ratio of expression was shown. (**B**) Inhibition of Hsp90 with 17-AAG reduced proteasome activity in A549 cells. Cells were treated with various concentrations of 17-AAG and 4 μM of MG-132 (positive control) for 24 h. Cells were washed with PBS and lysed with TNESV buffer without protease inhibitor. Cell lysate were analyzed for proteasome activity using synthetic fluorogenic peptide succinyl-Leu-Leu-Val-Tyr-7-amino-4-methylcoumarin. Statistical significance (p < 0.05) between experimental samples and the DMSO control is denoted by "*". (**C**) Inhibition of 26S proteasome increased the amount of survivin in A549 cells. A549 cells were treated with DMSO and various concentrations (1000 nM, 2000 nM, 4000 nM and 8000 nM) of the 26S proteasome inhibitor, MG-132, for 24 hours. The expression of survivin was revealed by Western blot analysis. Actin was used as an internal control. Relative ratio of expression was shown.

Survivin is normally degraded through the proteasomal degradation pathway and it has been shown that the 26S proteasome is responsible for this process [26]. On the other hand, Hsp90 plays a role in the assembly and maintenance of the 26S proteasome [20]. Furthermore, reduced proteasomal activity has been shown in 17-AAG and geldanamycin-treated cells [27,28]. Here, proteasome-dependent protein degradation pathway was investigated to determine whether proteasome plays a role in the reduced survivin degradation rate in 17-AAG treated A549, HONE-1 and HT-29 cells. In our study, Western blot analysis revealed that 17-AAG reduced the amount of 26S proteasome presented in A549 cells (Figure 6A). In addition, proteasomal activity assay also revealed that the activity of 26S proteasome was reduced by 25-30% in cells treated with 17-AAG (Figure 6B). To demonstrate that inhibition of 26S proteasome would subsequently affect the amount of survivin expressed in cells, the 26S proteasome inhibitor MG-132 was used. Western blot analysis clearly revealed that inhibition of the proteasomal activity by MG-132 induced survivin over-expression in a concentration-dependent manner (Figure 6C). Together with the results from the protein degradation experiment, these data suggest that increased survivin levels in A549 cells cannot simply be attributed to the induction of protein translation. Post-translational mechanisms such as the regulation of the 26S proteasome-dependent protein degradation may also play a role in survivin over-expression in 17-AAG treated A549 cells.

**Figure 7:**
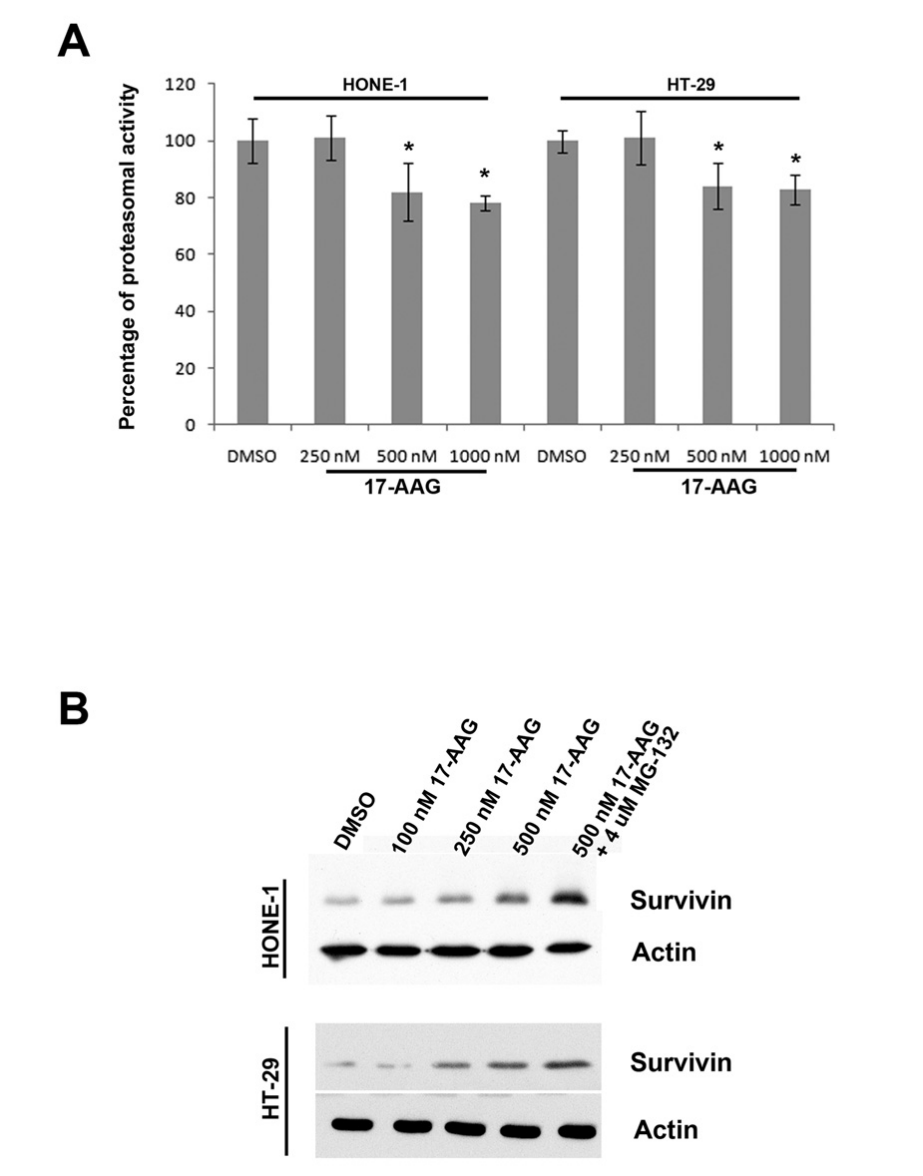
**Inhibition of Hsp90 with 17-AAG reduced proteasome activity in HONE-1 and HT-29 cells**. (A) Cells were treated with various concentrations of 17-AAG for 24 h. Cells were washed with PBS and lysed with TNESV buffer without protease inhibitor. Cell lysate were analyzed for proteasome activity using synthetic fluorogenic peptide succinyl-Leu-Leu-Val-Tyr-7-amino-4-methylcoumarin. Statistical significance (p < 0.05) between experimental samples and the DMSO control is denoted by "*". (**B**) Inhibition of 26S proteasome further increased the amount of survivin protein in 17-AAG treated HONE-1 and HT-29 cells. Cells were treated with DMSO and various concentrations (100 nM, 250 nM, 500 nM) of 17-AAG with/without MG-132 for 24 hours. The expression of survivin was revealed by Western blot analysis. Actin was used as an internal control.

To determine whether proteasomal activity was also affected in 17-AAG treated HT-29 and HONE-1 cells, proteasomal activity of drug-treated cells was measured. Proteasomal activity assay revealed that the activity of 26S proteasome was reduced by ~20% in HT-29 and HONE-1 cells treated with 17-AAG (Figure 7A). In addition, Western blot analysis revealed co-treatment of 17-AAG and the proteasome inhibitor, MG-132, induced synergistic increases in the level of survivin present in both cell lines (Figure 7B). These results indicated that the inhibition of 26S proteasome might also play a role in the up-regulation of survivin in 17-AAG treated HT-29 and HONE-1 cells.

### Targeting survivin increases drug sensitivity to 17-AAG in cancer cells

**Figure 8:**
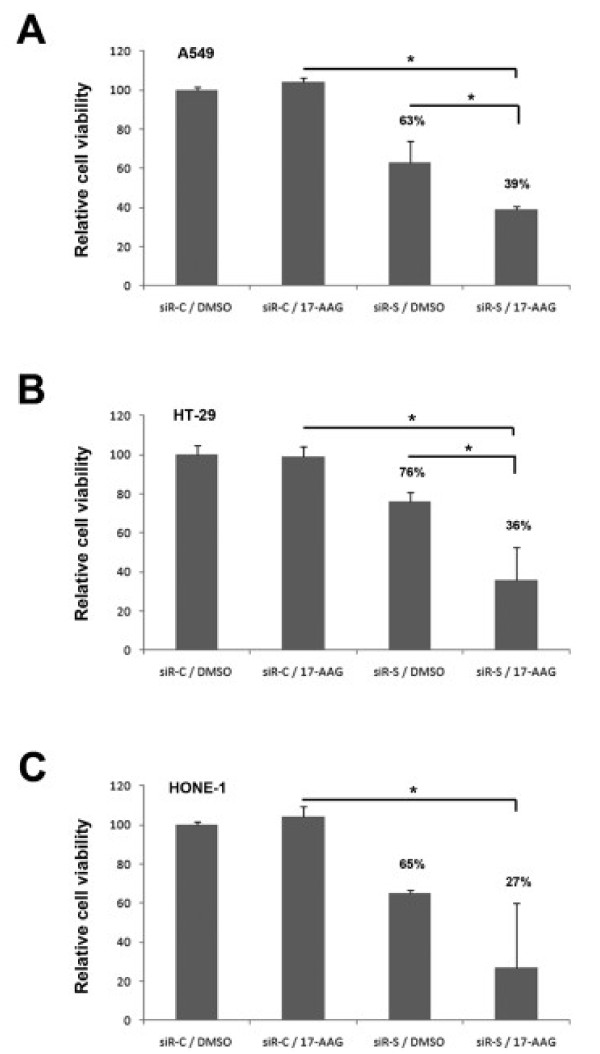
**Down-regulation of survivin enhanced the sensitivity to Hsp90 inhibitor in various cancer cells**. A549 (**A**), HT-29 (**B**) and HONE-1 (**C**) cells were transfected with siR-C (scramble control) and siR-S (survivin specific) siRNA oligomers for 48 h and subsequently incubated with/without 250 nM of 17-AAG for 24 h. MTT cell viability assay was used to determine the viability of various treatments treated cells. Statistical significance (p < 0.05) between experimental samples is denoted by "*".

It has been shown that silencing of survivin gene by small interfering RNAs produces supra-additive growth suppression in combination with 17-AAG in human prostate cancer cells [18]. To determine the functional importance of survivin in interfering with drug sensitivity to Hsp90 inhibitors in A549, HONE-1 and HT-29 cells, survivin was down-regulated by siRNA and cell viability was measured by MTT assay. Cells were treated with survivin-specific siRNA oligomer (siR-S) or scramble oligomer (siR-C) for 48 h and sub-subsequently incubated with/without 250 nM of 17-AAG for 24 h. Cells viability assay revealed that A549, HT-29 and HONE-1 cells treated with 250 nM of 17-AAG did not show reduced viability as compared to cells treated with DMSO (Figure 8). In addition, down-regulation of survivin by siR-S significantly reduced cell viability by ~30% in both cell lines as compared to cells transfected with control oligomer, siR-C (Figure 8). Interestingly, siR-S/17-AAG combination treatment further reduced the cell viability of A549, HT-29 and HONE-1 as compared to 17-AAG mono-treatment (Figure 8). Taken together, our results indicate that survivin plays an important role in the sensitivity to the Hsp90 inhibitor, 17-AAG, in our tested cancer cell lines.

## Discussion

It is widely believed that targeting Hsp90 with small molecule inhibitors is able to directly interfere with the physical interaction between Hsp90 and survivin, leading to the decrease of survivin protein level and induction of cancer cell death [5,15]. Interestingly, this study demonstrated for the first time that targeting Hsp90 with small molecule inhibitors will affect the expression of survivin at various stages, resulting in an increase of the amount of survivin protein presented in cancer cells. Furthermore, this study demonstrated that survivin plays an important role in the sensitivity to the Hsp90 inhibitor, 17-AAG, in cancer cells.

Here, we showed that targeting Hsp90 with small molecule inhibitor affected the amount of survivin mRNA transcript presented in cancer cells. It is not surprising that targeting Hsp90 induces different effect at the level of gene transcription in different cancer cells. Literatures revealed that the rate of survivin gene transcription is positively regulated by molecules such as sp1, sp3 and Myc [29,30]. In contrast, the gene transcription process of survivin is negatively regulated by molecules such as p53, retinoblastoma (Rb) and prostate-derived Ets transcription factor (PDEF) [31-33]. Importantly, Hsp90 interferes with sp1, sp3, p53 and Rb simultaneously [34,35]. Hence, differences in the response of survivin gene transcription may reflect different dependencies of various Hsp90-interfered and Hsp90-unrelated transcriptional factors on the expression of survivin in different cell types. Therefore, depending on the cellular context, targeting Hsp90 might indirectly up-regulate/down-regulate the process of survivin gene transcription through the interference with various survivin-related transcriptional factors.

Interestingly, our data also demonstrated that decreases at the mRNA level did not translate into decreases in survivin protein level in 17-AAG treated A549 cells. Together with results from the translation inhibition experiment, the protein degradation experiment and the examination of the survivin-related 26S proteasome, the current study strongly indicates that Hsp90 also interferes with survivin expression at the post-transcriptional level. Thus, Hsp90-targeted treatment interferes with the process of survivin gene transcription, protein translation and protein degradation simultaneously. In fact, Hsp90 plays an important role in the assembly and maintenance of the 26S proteasome [20,36]. The activity of 26S proteasome was shown to be reduced by the addition of the Hsp90 inhibitor, geldanaymicin, *in vitro *[20]. Reduced proteasomal activity was also shown previously in Hsp90-inhibited multiple myeloma cells [27]. On the other hand, previous studies demonstrated that indomethacin (NASID) and chlamydocin (HDAC inhibitor) enhanced survivin degradation through ubiquitin proteasome machinery in cells [37,38]. In our study, the use of proteasome inhibitor MG-132 was shown effective in increasing the amount of survivin present in our tested cancer cell lines, indicating that the activity of proteasome was important for survivin regulation. Therefore, the level of activity of proteasome might be one of the determinants of the amount of survivin present in Hsp90-inhibited cancer cells. However, it is hard to determine whether the interference with proteasome plays the most important role in the up-regulation of survivin. Further investigations are needed to determine the relative importance of transcription, translation and proteasome-related protein degradation in different Hsp90-targeted cancer cells. It is also worth noting that both 17-AAG and geldanamycin treatment reduced the amount of survivin presented in HeLa cells and this result was consistent with other studies. In contrast, results of the 3D-culture model revealed that 17-AAG treatment (1 μM) was also able to induce the over-expression of survivin in three dimensional cultured A549, HONE-1 and HT-29 cells (Additional file 1). Thus, the current study indicates that targeting Hsp90 may induce cell line-specific responses in the expression of survivin.

Importantly, results of the current study raise the concern that Hsp90 inhibitors might not function in a way as we previously thought. Indeed, literature reported that 17-AAG promoted formation of osteolytic lesions and bone metastases in murine breast cancer model, even though the drug reduced tumor growth at the orthotopic site [39]. Furthermore, Kayani *et al*. demonstrated that 17-AAG treatment was able to enhance the expression of Hsp70 in C2C12 muscle fiber cells and the recovery of extensor digitorum longus (EDL) following lengthening contraction-induced damage in animal model [40]. Thus, targeting Hsp90 with small molecular inhibitors may not be able to induce cell death in certain circumstances.

## Conclusion

In conclusion, the current study reveals the complex interaction between Hsp90 and survivin in cancer cells. Besides stabilizing the survivin protein through simple physical interaction, Hsp90 also indirectly interferes with survivin expression through transcription, translation and proteasome-related protein degradation. These novel findings suggest a model in which gene transcription, together with protein translation and proteasomal degradation, constitute a platform capable of modulating the amount of survivin expressed in Hsp90-targeted cancer cells. Our findings suggest that down-regulation of survivin is not a definitive therapeutic function of Hsp90 inhibitors and that dual inhibition of Hsp90 and survivin may be warranted.

## Materials and methods

### Cell lines, antibodies and reagents

The human lung carcinoma (A549), nasopharyngeal carcinoma (HONE-1) and colorectal adenocarcinoma (HT-29) cells were purchased from the American Type Culture Collection (ATCC, Manassas, VA). A549 cells were cultured in RPMI 1640 medium (Gibco, Grand Island, NY), supplemented with 10% fetal bovine serum, penicillin (100 U/mL), streptomycin (100 μg/mL) and L-glutamine (0.29 mg/mL), at 37°C. HONE-1 and HT-29 cells were cultured in RPMI 1640 medium (Gibco, Grand Island, NY), supplemented with 5% fetal bovine serum, penicillin (100 U/mL), streptomycin (100 μg/mL) and L-glutamine (0.29 mg/mL), at 37°C. The antibodies used in this study included a mouse anti-Actin antibody (Santa Cruz Biotechnology, Santa Cruz, CA), a rabbit anti-Survivin antibody (R&D Systems, Minneapolis, MN), a rabbit anti-Akt antibody (Cell Signaling Technology, Danvers, MA) and a mouse anti-26S proteasome antibody (abcam, Cambridge, UK). Hsp90 inhibitors used in this study included: 17-AAG (Calbiochem, Darmstadt, Germany), geldanamycin (Calbiochem, Darmstadt, Germany) and cycloheximide (Calbiochem, Darmstadt, Germany).

### Real-time reverse transcription-polymerase chain reaction (Real-time PCR)

Expression level of survivin transcript was determined by real-time reverse transcriptase (RT)-polymerase chain reaction (PCR) using a LightCycler instrument (Roche, Indianapolis, IN). Primers and Taqman probes were designed by Probe Finder™ http://www.universalprobelibrary.com. Taqman probes were from the Universal Probe Library: survivin and hGAPDH. Specific primers with following sequences were used: survinin forward, 5' GCCCAGTGTTTCTTCTGCTT; Survivin reverse, 5'CCGGACGAATGCTTTTTATG; hGAPDH forward, 5' AGCCACATCGCTCAGACAC and hGAPDH reverse, 5' GCCCAATACGACCAAATCC. The real-time PCR condition was as follows: 1 cycle of initial denaturation at 95°C for 10 min, 45 cycles of amplification at 95°C for 10 s, 60°C for 30 s, and 72°C for 1 s, with a single fluorescence acquisition. hGAPDH gene was used as an internal control. All experiments have been repeated twice.

### SDS-PAGE and Western blot analysis

Cells were lysed with ice-cold lysis buffer (10 mM Tris, 1 mM EDTA, 1 mM DTT, 60 mM KCl, 0.5% NP-40 and protease inhibitors). Total cell lysates, fractions of supernatant or pellet were resolved on 10% and 12% polyacrylamide SDS gels under reducing conditions. The resolved-proteins were electrophoretically transferred to PVDF membranes (Amersham Life Science, Amersham, U.K.) for Western blot analysis. The membranes were blocked with 5% non-fat milk powder at room temperature for two hours, washed twice with PBST (1% Tween) and then incubated with primary antibody for 90 minutes at room temperature. The membranes were washed twice with PBST then subsequently incubated with a horseradish peroxidase-conjugated secondary antibody (dilution at 1:10000, Santa Cruz Biotechnology, Santa Cruz, CA). Immunoreactivity was detected by Enhanced Chemiluminescence (Amersham International, Buckingham, U.K.) and autoradiography. All experiments have been repeated twice.

### Proteasome activity assay

Cells exposed to various concentrations of 17-AAG and MG-132 for 24 h were washed twice with PBS and lysed with TNESV buffer [50 mM Tris-HCl (pH 7.5), 1% NP40 detergent, 2 mM EDTA, 100 mM NaCl, 10 mM sodium orthovanadate] without protease inhibitors. Cell lysate were assayed for proteasome chymotrypsin activity using the synthetic fluorogenic peptide chymotrypsin substrate, N-Succinyl-Leu-Leu-Val-Tyr-AMC. Fluorescent signals were measured with a 96-well plate reader with an excitation wavelength of 380 nm and emission wavelength of 460 nm. All experiments have been performed as triplicate and repeated twice.

### siRNA

Target-validated siRNA oligos (Santa Cruz Biotechnology, Santa Cruz, CA) were transfected into cells using the Lipofectamine-2000 reagent (Invitrogen, Carlsbad, CA). Briefly, cells were seeded onto 96-well plates or chamber-slides, and cultured overnight in 100 μl of antibiotic-free RPMI media. siRNA oligomers (8 pmol in 0.4 μl) were diluted in 25 μl of Opti-MEM^® ^I medium (Invitrogen, Carlsbad, CA) without serum, and then mixed with 0.2 μl of Lipofectamine-2000 transfection reagent for 25 min at room temperature. Cells were overlaid with the transfection mixture, and incubated for various times.

### MTT cell viability assay

Cells seeded onto 96-well plates were transfected with/without survivin-specific siRNA oligomer for 48 h and subsequently treated with 17-AAG for 24 h. 25 μl of MTT (5 mg/mL) was added to each sample and incubated for 4 hours, under 5% CO_2 _and 37°C. 100 μl of lysis buffer (20% SDS, 50% DMF) was subsequently added into each sample and further reacted for 16 hours.

## Abbreviations

**17-AAG**: 17-(Allylamino)-17-demethoxygeldanamycin; **Hsp90**: heat-shock protein 90; **siR-C**: scramble siRNA oligos; **siR-S**: survivin-targeted siRNA oligos.

## Competing interests

The authors declare that they have no competing interests.

## Authors' contributions

CHAC performed most of the *in vitro *studies and drafted the manuscript. HHC performed the quantitative RT-PCR analysis. LTC participated in sample preparation and also revised the manuscript. KWL participated in sample preparation and a few preliminary experiments. JRK provided HeLa cells and re-confirmed some of the experimental results. JYC coordinated the study. All authors read and approved the final manuscript.

## Authors' information

C. H. A. Cheung, *Ph.D*. (*Post-doctoral research fellow, molecular biologist*)

H. H. Chen, *Ph.D*. (*Post-doctoral research fellow, molecular biologist*)

L.T. Cheng, *Ph.D*. (*Post-doctoral research fellow, molecular biologist*)

K.W. Lyu, *M.Sc. (Research student)*

J.R. Kanwar, *Ph.D. (Principle investigator, Assistance professor, molecular biologist)*

J. Y. Chang, *M.D. (Distinguished investigator, Professor, medical oncologist)*

## Supplementary Material

Additional file 1 **17-AAG treatment induced the over-expression of survivin in three-dimensionally cultured A549, HONE-1 and HT-29 cells.** HeLa, A549, HONE-1 and HT-29 cells were three-dimensionally cultured in the RPMI/Matrigel^® ^matrix for five days and subsequently treated with 1 μM of 17-AAG for 24 hours. Expression of survivin was analyzed by Western blot analysis. (**A**) Various 3D-cultured cancer cells were shown by light microscopy. (**B**) Western blot analysis revealed that 17-AAG treatment induced the over-expression of survivin in 3D-cultured A549, HONE-1 and HT-29 cells. In contrast, the same treatment reduced the expression of survivin in HeLa cells. Click here for file
