# Supplementary figures and images for: Targeting Hsp90 with small molecule inhibitors induces the over-expression of the anti-apoptotic molecule, survivin, in human A549, HONE-1 and HT-29 cancer cells
Source: Mol Cancer. 2010 Apr 15;9:77. doi: 10.1186/1476-4598-9-77 (PMC2873435; doi:10.1186/1476-4598-9-77)

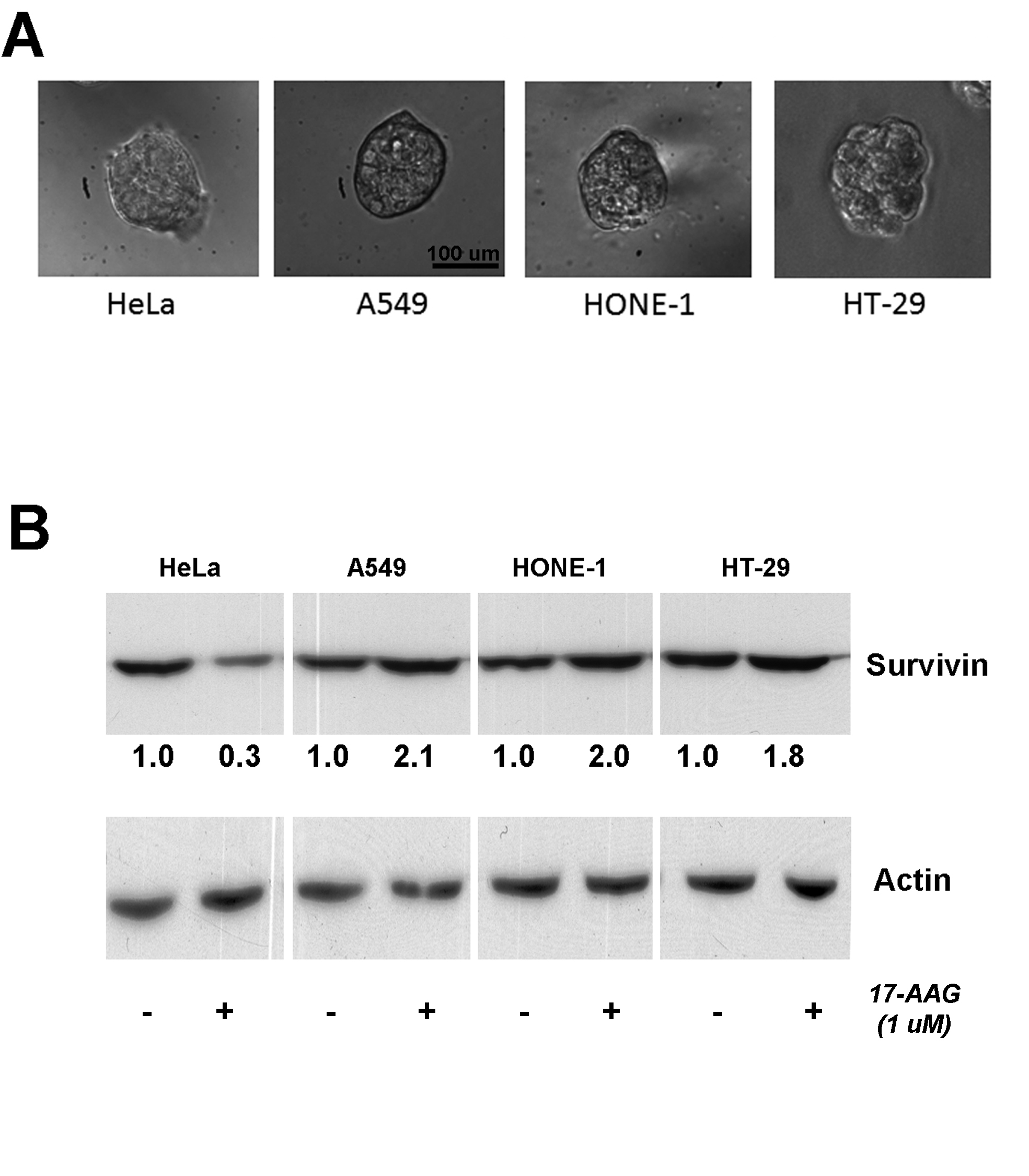

Supplement: Additional file 1 — 17-AAG treatment induced the over-expression of survivin in three-dimensionally cultured A549, HONE-1 and HT-29 cells. HeLa, A549, HONE-1 and HT-29 cells were three-dimensionally cultured in the RPMI/Matrigel® matrix for five days and subsequently treated with 1 μM of 17-AAG for 24 hours. Expression of survivin was analyzed by Western blot analysis. (A) Various 3D-cultured cancer cells were shown by light microscopy. (B) Western blot analysis revealed that 17-AAG treatment induced the over-expression of survivin in 3D-cultured A549, HONE-1 and HT-29 cells. In contrast, the same treatment reduced the expression of survivin in HeLa cells. [file 1476-4598-9-77-S1.JPEG]
